# Complex I Function and Supercomplex Formation Are Preserved in Liver Mitochondria Despite Progressive Complex III Deficiency

**DOI:** 10.1371/journal.pone.0086767

**Published:** 2014-01-22

**Authors:** Mina Davoudi, Heike Kotarsky, Eva Hansson, Vineta Fellman

**Affiliations:** 1 Department of Pediatrics, Clinical Sciences, Lund University, Lund, Sweden; 2 Folkhälsan Research Center, Helsinki, Finland; 3 Children’s Hospital, Helsinki University Hospital, University of Helsinki, Helsinki, Finland; University of Florida, United States of America

## Abstract

Functional oxidative phosphorylation requires appropriately assembled mitochondrial respiratory complexes and their supercomplexes formed mainly of complexes I, III and IV. BCS1L is the chaperone needed to incorporate the catalytic subunit, Rieske iron-sulfur protein, into complex III at the final stage of its assembly. In cell culture studies, this subunit has been considered necessary for supercomplex formation and for maintaining the stability of complex I. Our aim was to assess the importance of fully assembled complex III for supercomplex formation in intact liver tissue. We used our transgenic mouse model with a homozygous c.232A>G mutation in *Bcs1l* leading to decreased expression of BCS1L and progressive decrease of Rieske iron-sulfur protein in complex III, resulting in hepatopathy. We studied supercomplex formation at different ages using blue native gel electrophoresis and complex activity using high-resolution respirometry. In isolated liver mitochondria of young and healthy homozygous mutant mice, we found similar supercomplexes as in wild type. In homozygotes aged 27–29 days with liver disorder, complex III was predominantly a pre-complex lacking Rieske iron-sulfur protein. However, the main supercomplex was clearly detected and contained complex III mainly in the pre-complex form. Oxygen consumption of complex IV was similar and that of complex I was twofold compared with controls. These complexes in free form were more abundant in homozygotes than in controls, and the mRNA of complex I subunits were upregulated. In conclusion, when complex III assembly is deficient, the pre-complex without Rieske iron-sulfur protein can participate with available fully assembled complex III in supercomplex formation, complex I function is preserved, and respiratory chain stability is maintained.

## Introduction

In intact mitochondria, the respiratory chain consists of appropriately assembled complexes, which are further arranged in supercomplexes, also called respirasomes or supramolecular formations [1]. In mammals, supercomplexes contain mainly complexes I, III, and IV (CI, CIII and CIV) in different stoichiometric combinations [2], [3]. Supercomplexes have functional importance, since they stabilize the levels of individual complexes [4], [5], enhance the efficiency of electron transfer through them by substrate channelling [6] and control the generation of reactive oxygen species (ROS) [7]. Supercomplex formation is dependent on the presence of phospholipids [8] and is facilitated by recently identified supercomplex assembly factors in yeast [9], [10], [11] and mammals [12], [13].

A recent human cancer cell study assessing respirasome assembly after reversible inhibition of mitochondrial translation suggested that CI plays a central role in the formation of supercomplexes [14]. A CI assembly intermediate would serve as a scaffold for incorporating CIII and CIV before addition of the NADH dehydrogenase module (that includes the subunit NDUFV1) to finalize respirasome assembly. According to this model, assembly of holo-CIII and -CIV and their association with the CI subassembly are two necessary steps for biogenesis of the respirasome [14].

We addressed the problem of supercomplex assembly *in vivo* using a transgenic mouse model, in which a homozygous mutation (c.232A>G) in the CIII chaperone gene *Bcs1l* causes progressive CIII deficiency due to decreasing incorporation of the Rieske iron-sulfur protein (RISP) subunit into CIII [15]. Mutations in the human *BCS1L* gene are major causes of disorders with CIII deficiency [16]. Depending on the mutation site in *BCS1L* and additional unknown factors, the resulting phenotypes vary considerably, the most severe being a lethal neonatal disorder, the GRACILE syndrome (Fellman disease, MIM 603358) [17], [18]. Homozygous mice with this mutation (*Bcs1l^G/G^*) are initially symptom-free but after three weeks of age develop progressive fatal hepatopathy, mimicking the human disease, in parallel with decreased incorporation of RISP into CIII and progressive functional deficiency of CIII [15]. This model presents an opportunity to investigate the importance of RISP incorporation into CIII for supercomplex formation.

We hypothesized that incompletely assembled CIII leads to disturbed supercomplex formation and functional deficiency in mitochondria. Thus, we assessed the size and abundance of supercomplexes and the activities of CI, CIII and CIV as RISP was progressively depleted from CIII in *Bcs1l^G/G^* mouse liver mitochondria.

## Materials and Methods

### Animal Experiments

*Bcs1l^G/G^* and control mice [15], more than 99% congenic C57Bl/6, were maintained on rodent diet (Labfor R34, Lactamin, Stockholm, Sweden) and water ad libitum in a vivarium with 12 h light/dark cycle at 22°C. Littermate wild type (*Bcs1l^A/A^*) or heterozygous (*Bcs1l^A/G^*) mice that are phenotypically similar to wild type [15] were used as controls. Animals were studied at different ages; young and healthy aged 14 and 16 days (P14, P16), at P20–22 when the first signs of liver histopathology are found, and at P26–P29 when clear liver disease appears [15]. The animals were sacrificed by cervical dislocation, and tissues collected immediately after death. Tissue samples were used for isolation of mitochondria or frozen on dry ice and stored at −80°C until use.

### Ethics Statement

Animal experiments were performed with the approval of the Lund regional animal research committee, Sweden (permits, M 253-08, M 274-10, 31-8265/08) according to national guidelines. All efforts were taken to ameliorate suffering.

### Preparation of Mouse Liver Mitochondria

Mouse liver tissue was collected in ice cold isolation buffer (320 mM Sucrose, 10 mM Trizma Base, 2 mM EGTA) and subsequently homogenized in 2 ml isolation buffer supplemented with 0.1% BSA. Mitochondria were prepared from homogenates by sequential centrifugation including density purification on 19% Percoll (GE Healthcare, Amersham, UK) [19], [20]. The amount of mitochondria was measured as protein absorbance at 280 nm on a Nanodrop (Fisher Scientific, Gothenburg, Sweden). Isolated mitochondria were either used directly or aliquoted and stored at −80°C.

### Isolation of Respiratory Chain Complexes and Supercomplexes

Frozen mitochondrial pellets were dissolved in phosphate buffered saline (PBS) supplemented with Complete Mini Protease Inhibitor (Roche Diagnostics Scandinavia AB, Stockholm, Sweden), and the mitochondrial protein concentrations were measured using Nanodrop (Fisher Scientific, Gothenburg, Sweden). Mitochondria were pelleted for 5 min at 5000 g and subsequently dissolved to a concentration of 5 mg protein/ml in MB2 buffer (1.75 M aminocaproic acid, 75 mM Bis-Tris, pH 7.0, 2 mM EDTA, pH 8.0). Mitochondrial membrane proteins were solubilized by incubation with 0.8% digitonin (Sigma Aldrich, Stockholm, Sweden) for 5 min on ice. Samples were centrifuged for 30 min at 13000 g, the supernatant was collected and the protein concentration measured as before. Finally, SBG (750 mM aminocaproic acid, 5% Serva Blue G from SERVA Electrophoresis GmbH, Heidelberg, Germany) was added to a final concentration of 4.5%. These samples were stored at −80°C for electrophoresis.

### SDS PAGE, Blue Native Gel Electrophoresis (BNGE), 2-dimensional BNGE and Western Blot

Five µg mitochondrial membrane proteins were separated by either 10% SDS PAGE or Blue Native Bis-Tris PAGE 4–16% (Invitrogen, Carlsbad, CA, USA). For 2-dimensional (2D) BNGE, strips representing the first dimension BNGE lanes were cut, incubated with 1% β-mercaptoethanol and 1% SDS and overlaid on 10% SDS PAGE [21]. Gels were blotted onto polyvinylidine difluoride membranes using iblot™ dry equipment (Invitrogen, Carlsbad, CA, USA) and membranes were blocked in PBS supplemented with 0.05% Tween 20 and 5% dry milk for subsequent antibody incubation. Almost all antibodies for detection of respiratory chain subunits were obtained from MitoSciences (Eugene, Oregon, USA). The following antibodies were used: CI subunit NDUFA9 (MS111), CII subunit 30 kDa (MS203), CIII subunits Rieske (MS305) detecting fully assembled CIII and Core1 (MS303) detecting both precomplex and fully assembled CIII, CIV subunit I (MS404) and subunit Va (MS409), and OXPHOS mix (MS603) detecting CI subunit NDUFA9, CII 70 kDa subunit, CIII subunit Core2, CIV subunit IV, and CV subunit alpha, as well as Porin (MSA05), ETFAα (MS782), PDHE1α (MSP07) and GAPDH (9484 from Abcam, Cambridge, UK). We used NDUFV1 antibody (Sigma Aldrich, Stockholm, Sweden) for detection of fully assembled CI, and BCS1L antibody from Abnova (Taipei, Taiwan). Primary antibodies were detected by incubation with HRP-coupled goat anti-mouse secondary antibody (DAKO Cytomation, P0447). Membranes were developed with ECL plus (GE Healthcare, Amersham, UK). Exposure time was 1–10 min.

### High Resolution Respirometry

Mitochondrial oxygen consumption in freshly isolated mitochondria (protein concentration 250 mg/ml) was measured using an Oroboros Oxygraph-2k with DatLab 4 software (Oroboros Instruments, Innsbruck, Austria). Experiments were run at 37°C in mitochondrial respiration medium MIR05 supplemented stepwise with substrates and inhibitors for individual complexes, using the SUIT protocol as previously published [15], [22]. Maximal capacity of the respiratory chain was obtained by titrating with FCCP. Inhibition of CI by rotenone revealed the contribution of CII, and finally addition of antimycin-A determined the residual oxygen consumption (Table 1).

**Table 1:** Respiratory chain function, measured as oxygen consumption (O_2_ flux/pmol/(s x mg), mean±SEM) with high resolution respirometry on isolated liver mitochondria using SUIT protocol [19] with sequential addition of substrates, ADP, and inhibitors, showed significant changes in sick 27–29 days *Bcs1l^G/G^* animals compared to control animals (*Bcs1l^A/A^*).

SUIT sequence	Substrate forindicated complex	Inhibitor ofindicated complex	*Bcs1l ^A/A^* (n = 4)	*Bcs1l ^G/G^* (n = 4)	p value
Basal respiration			3.8±4.6	7.5±3.2	0.33
Malate/Pyruvate	CI		24±3.9	58±7.0	**<0.001**
ADP			138±23	179±52	0.13
Glutamate	CI		175±23	209±51	0.21
Succinate	CII		338±78	201±45	**0.04**
Oligomycin		CV	85±13	60.5±4.5	0.05
FCCP*			727±54	488±158	**0.02**
Rotenone		CI	563±91	379±102	0.08
Antimycin		CIII	16.4±9.2	8.0±1.7	0.14
TMPD**	CIV		1590±163	1723±312	0.54

* FCCP, Carbonyl cyanide-*4*-(trifluoromethoxy)phenylhydrazone, uncoupler showing maximal capacity.

** TMPD *N,N,N′,N′*-tetramethyl-*p*-phenylenediamine.

### RNA Preparation and Quantitative PCR

RNA was extracted from snap frozen mouse liver tissue using the RNeasy Mini kit and RNase free DNase set according to the manufacturer’s recommendations (Qiagen GmbH, Düsseldorf, Germany), and RNA quantity and quality were analyzed with Nanodrop and gel. For quantification RNA was reverse transcribed using Taqman® reverse transcription reagents from Applied Biosystems (Invitrogen, Carlsbad, CA, USA). The resulting cDNA was used as template in real time reactions on a StepOne platform using the Taqman® Gene Expression assays Mm00518001_m1, Mm00841715_m1, Mm01205647_g1, and Mm03302249_g1, Mm00481849_m1, Mm00445911_m1, Mm00445961_m1, Mm00481216_m1, Mm00504941_m1, Mm00458272_m1, Mm00432638_m1, Mm01259143_g1 from Applied Biosystems (Invitrogen, Carlsbad, CA, USA). Expression values were normalized against the housekeeping gene *Gapdh* (Mm9999915_g1).

### Statistics

The data are presented as median (densitometry) or mean ± SEM for respirometry and quantitative PCR. Group differences were analyzed with t-test using Graph Pad Prism 5 software. Respirometry results were analyzed with paired t-test as previously described [15]. P-values <0.05 were considered significant.

## Results

### Intracellular Localization of RISP in *Bcs1l^G/G^* Mice with CIII Deficiency

**Figure 1:**
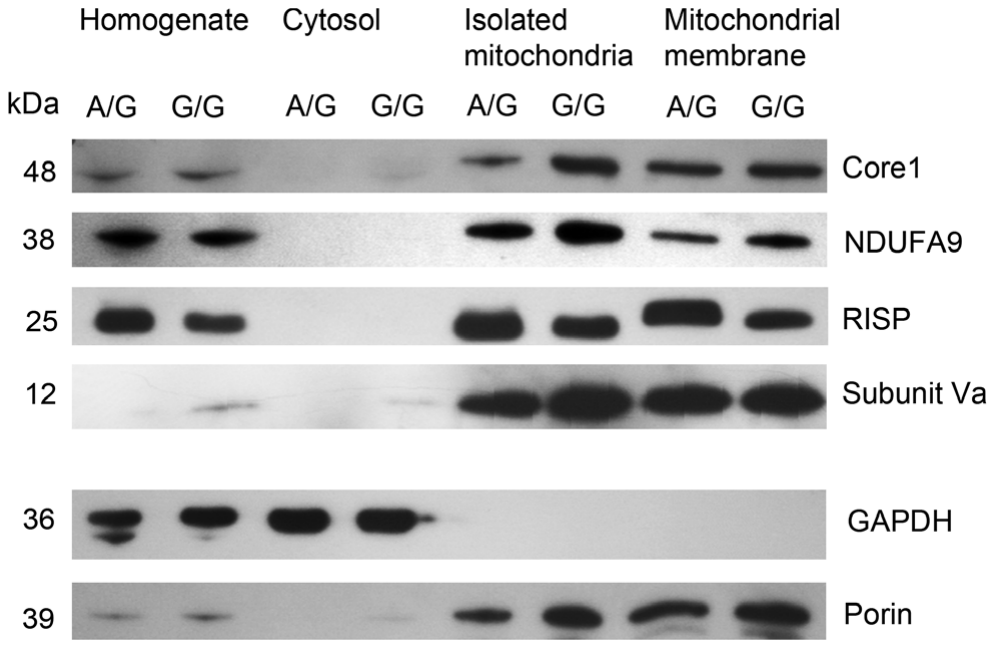
Mitochondria from *Bcs1l^G/G^* mice contain less membrane located RISP. Liver tissue from sick *Bcs1l* ^G/G^ (G/G) and control (A/G) mice aged P26 was homogenized and subsequently separated into cytosolic, mitochondrial and mitochondrial membrane fractions. These fractions were analyzed by SDS PAGE and Western blot detecting CIII subunits Core1 and RISP, CI subunit NDUFA9, and CIV subunit Va. Porin and Glyceraldehyde-3-phosphate dehydrogenase (GAPDH) were used as markers for mitochondrial membrane and cytosol, respectively, and as loading controls.

Using SDS PAGE and Western blot, we assessed RISP content in liver homogenates, hepatocyte cytosols, isolated mitochondria, and mitochondrial membrane preparations of *Bcs1l^G/G^* mice aged 26 days (Figure 1). In liver homogenates and isolated mitochondria, RISP content was somewhat diminished compared to control animals, and in mitochondrial membrane preparations the band was clearly decreased. The bands representing CIII subunit Core1, CI subunit NDUFA9, and CIV Subunit Va in isolated mitochondria and mitochondrial membranes were similar to controls (Figure 1).

### RISP Content as a Function of Age in *Bcs1l^G/G^* Mice

**Figure 2:**
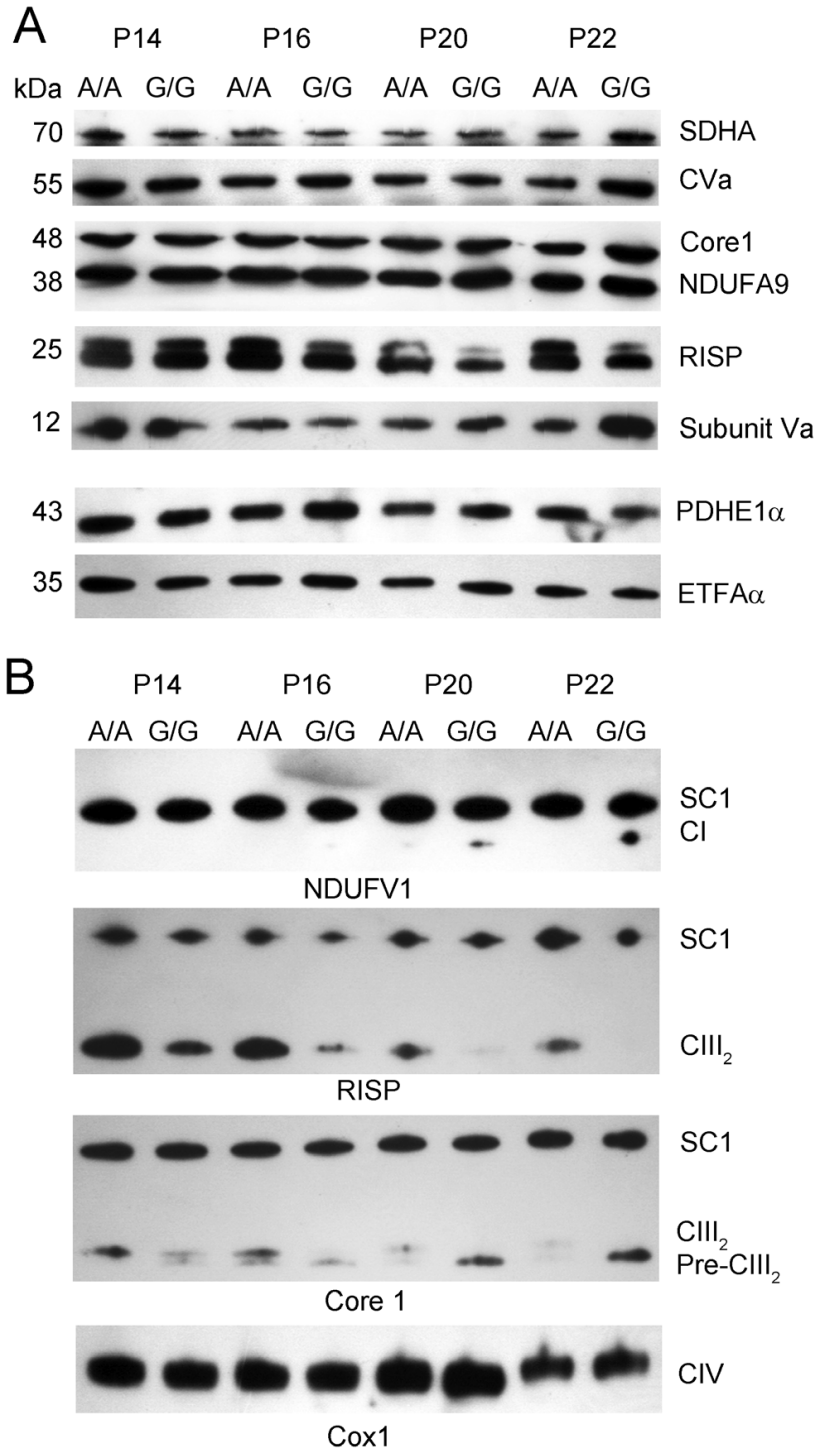
Age dependent decrease of RISP in young *Bcs1l* ^G/G^ mice. A. Subunits of all complexes were assessed after SDS PAGE of isolated mitochondria from *Bcs1l* ^G/G^ (G/G) and wild type (A/A) young mice using the following antibodies; SDHA (CII), CVa (CV), Core1 (CIII), NDUFA9 (CI), RISP (CIII), and Subunit Va (CIV). Pyruvate dehydrogenase (PDHE1α) and electron-transfer-flavoprotein, alpha polypeptide (ETFAα) were used as loading controls. B. Supercomplexes in homozygous and wild type young mice were investigated with BNGE and Western blot using antibodies detecting subunits NDUFV1, RISP, Core1 and subunit Cox1 (CIV).

To elucidate potential secondary effects of RISP deficiency in CIII on the other respiratory chain complexes, we analyzed complex subunits in mitochondria with SDS PAGE. In wild type animals, RISP content was similar at ages P14 to P22. In contrast, in *Bcs1l^G/G^* mice from P16 onward, a progressive decrease of RISP protein developed (Figure 2A). The decreasing RISP amount in *Bcs1l^G/G^* mice was not accompanied by any significant change in the amount of other complex subunits at age P14 to P22 (Figure 2A).

### Supercomplex Formation in *Bcs1l^G/G^* Mice

Next, we investigated whether the progressive decrease of RISP in CIII of *Bcs1l^G/G^* mice influences the formation of other respiratory chain complexes and supercomplexes. In isolated liver mitochondria of mice aged P14 to P22, using BNGE and Western blot we found one major supercomplex (SC1) composed of CI and CIII (Figure 2B). Immunoblotting with the late assembly subunit NDUFV1 showed that CI was fully assembled in SC1 in mitochondria of both *Bcs1l^G/G^* and wild type mice (Figure 2B). At ages P14–16, in neither group free CI was found, but from age P20 free CI appeared in *Bcs1l^G/G^* mice.

In control animals using RISP and Core1 antibodies, fully assembled CIII was detected at all ages in SC1 and as a CIII dimer (CIII_2_) that diminished with increasing age (Figure 2B). In mutant mice, the RISP containing CIII_2_ was less abundant at all ages diminishing to non-detectable level at P22. This was accompanied by a subsequent decrease of fully assembled CIII in SC1 (Figure 2B). No differences were found between the young homozygous and wild type mice in CIV abundance detected with subunit Cox1 (Figure 2B).

**Figure 3:**
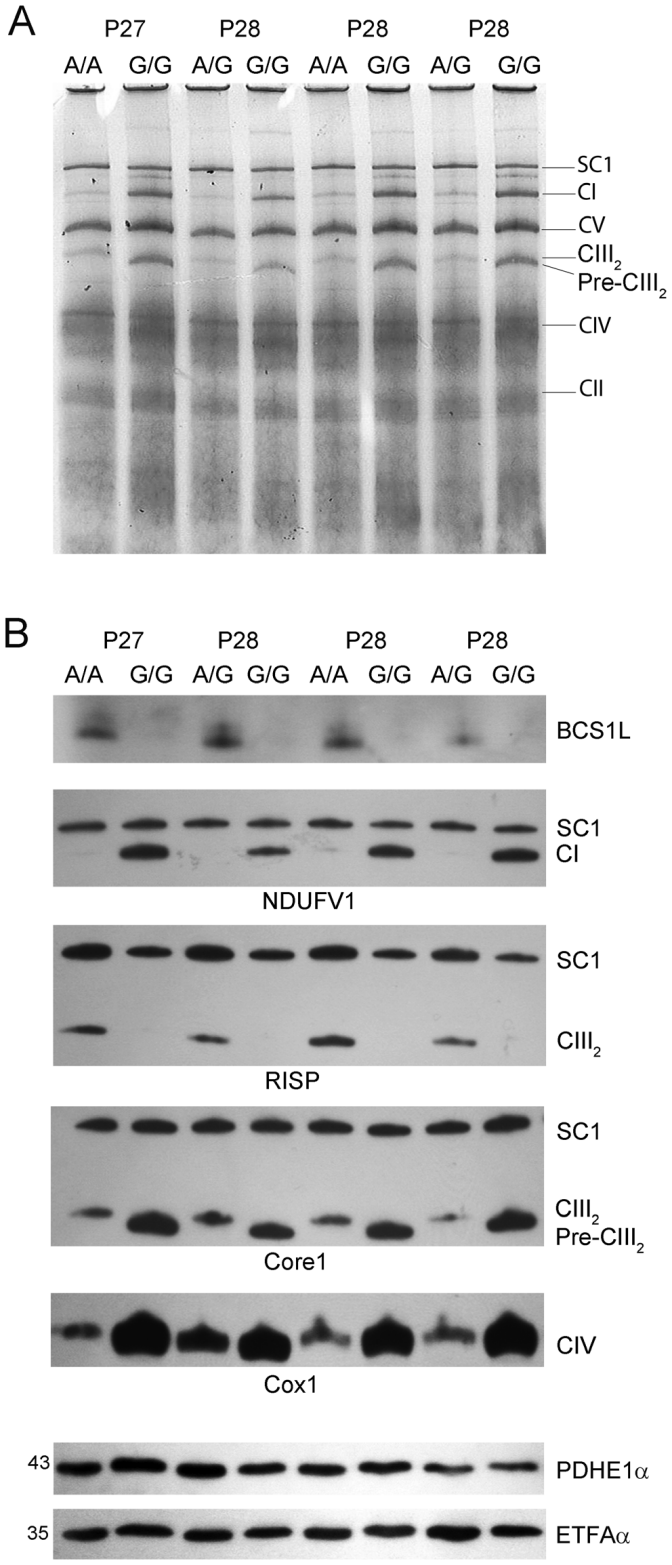
Low amount of RISP in sick *Bcs1l* ^G/G^ mice is associated with increased amount of free pre-CIII_2_ and CI. A. Representative blue native gel electrophoresis of isolated mitochondria from *Bcs1l* ^G/G^ (G/G) and control (A/A and A/G) mice 27–28 days old. The bands of respiratory chain complexes and supercomplexes are visualized. B. In isolated liver mitochondria from *Bcs1l* ^G/G^, BNGE followed by Western blot shows the lack of BCS1L. Supercomplex (SC1) composition was assessed with antibodies against NDUFV1, RISP, Core1 and subunit Cox1. PDHE1α and ETFAα antibodies were used as loading controls.

With BNGE we determined the relative sizes of complexes and supercomplexes in 4 pairs of mice aged 27–28 days, and found that CIII was present in isolated mitochondria from sick homozygotes as a dimer with a slightly smaller molecular weight than in wild type animals (Figure 3A). The CI band was clearly denser than in controls. In immunoblots using the NDUFV1 subunit, we found abundant fully assembled CI in free form in mutant homozygotes, whereas free CI was almost undetectable in control animals (Figure 3A,B). Fully assembled CI was present in comparable amounts in SC1 in mitochondria from *Bcs1l^G/G^* and control mice (Figure 3B).

In sick homozygotes, BCS1L protein had almost completely disappeared (Figure 3B). Previously we have shown decreased BCS1L content at all ages compared to controls [15]. The missing BCS1L in homozygotes was accompanied by lack of RISP in CIII_2_ and accumulation of free pre-complex CIII (pre-CIII_2_) without RISP, shown with the Core1 antibody. It detected the complex as a band with a slightly smaller molecular weight than in control animals (Figure 3B), also explaining the smaller CIII size in BNGE (Figure 3A). Completely assembled CIII detected with RISP antibody was only located in SC1, which was clearly decreased compared to control animals (Figure 3B). Quantification (n = 4 per group, Figure 3B) by densitometry showed that median RISP content in SC1 of homozygotes was 65% of controls (p = 0.02). The Core1 content in SC1 was similar in both groups. With Cox1 antibody more CIV was found in mutant than in control mitochondria (Figure 3B).

### Supercomplex Composition in *Bcs1l^G/G^* Mice

To identify possible hidden epitopes, we performed 2D-BNGE on isolated liver mitochondria to separate the subunits of complexes and supercomplexes, and identified them with Western blot.

**Figure 4:**
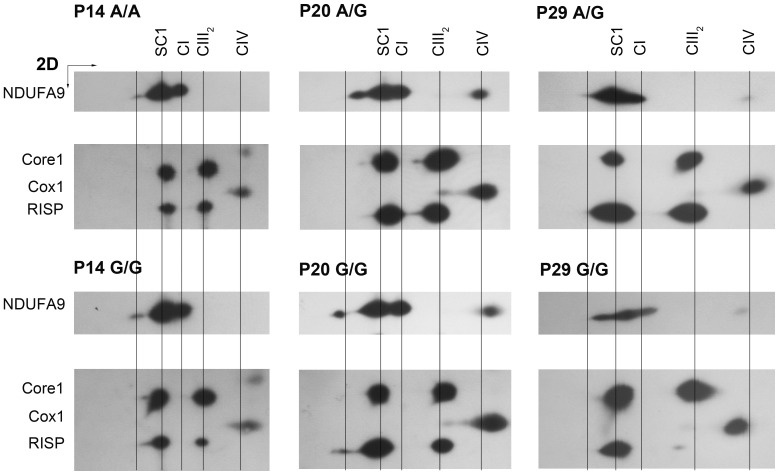
The main supercomplex contains CI, pre-CIII and fully assembled CIII_2_. Isolated liver mitochondria from P14, P20 and P29 *Bcs1l* ^G/G^ (G/G) and control (A/A and A/G) mice were analyzed with two-dimensional run with BNGE/SDS PAGE and Western blot. Antibodies for complexes are the same as in Figure 2 and 3. The ratio of RISP/Core1 was clearly smaller in P29 homozygotes than in control animals.

In control and homozygous mice of all ages, the CI subunit NDUFA9 was present in two distinct bands (Figure 4) corresponding to SC1 and CI in BNGE with SC1 being the most prominent in control animals (Figure 4). In SC1 of *Bcs1l^G/G^*, less NDUFA9 and RISP were detected with increasing age, but Core1 was abundant (Figure 4) and as also shown in Figure 3B increased in free form at P29 compared to controls. CIV was not detected in SC1.

### Respiratory Chain Function in Liver Mitochondria of *Bcs1l^G/G^* Mice

Oxygen consumption in isolated mitochondria from P27–29 animals showed that basal respiration was slightly, but not significantly, elevated in *Bcs1l^G/G^* compared with controls, and that respiration increased more after addition of the CI substrates malate and pyruvate, followed by ADP (Table 1). However, subsequent addition of the CII substrate succinate resulted in significantly lower oxygen consumption in *Bcs1l* ^G/G^ mitochondria than in wild type (Table 1). The maximal electron transport capacity assessed by addition of the uncoupler FCCP was lower in *Bcs1l^G/G^* mitochondria compared with controls. CIV oxygen consumption with CIV substrate TMPD was similar in both groups (Table 1). Taken together, despite verified diminished CIII activity in *Bcs1l^G/G^* mitochondria [15], oxygen consumption based on CI substrates was somewhat increased compared with controls.

### Expression of *Bcs1l* and Complex Subunits in *Bcs1l^G/G^* Liver

**Figure 5:**
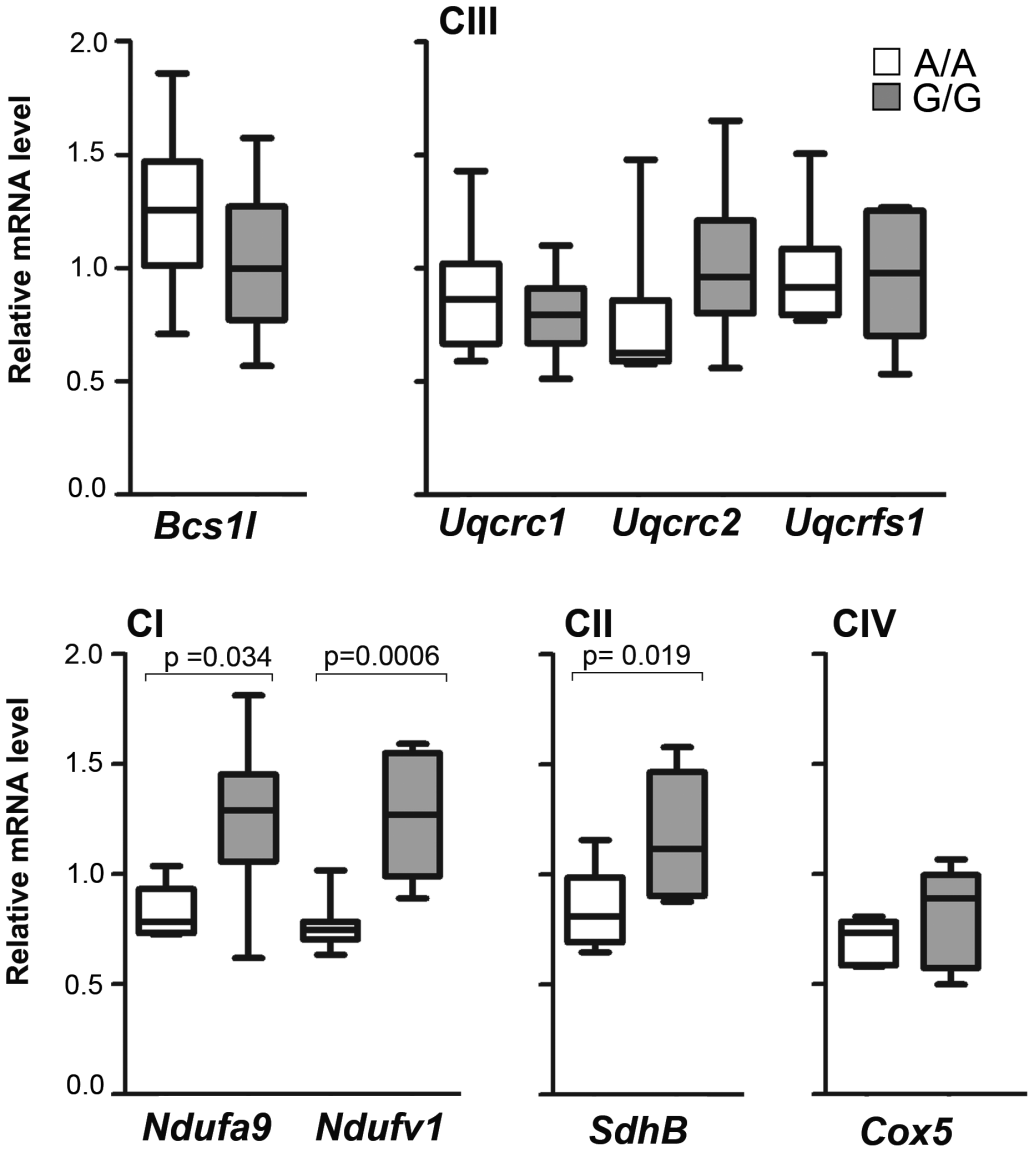
Increased expression of CI and CII subunits in liver tissue of sick *Bcs1l* ^G/G^ animals. The mRNA expression levels of *Bcs1l* and subunits of respiratory chain complexes were analyzed with quantitative PCR. In homozygotes, the subunits of CI and that of CII were significantly up-regulated in comparison with control animals.

In sick homozygotes (n = 6), mRNA levels of *Bcs1l* and the CIII subunits Core1 (*Uqcrc1*), Core2 (*Uqcrc2*) and RISP (*Uqcrfs1*) were comparable to those of control animals (A/A n = 6 and A/G n = 2), whereas increased expression was found for CI and CII subunits (Figure 5).

## Discussion

In our homozygous mutant mice, in which RISP incorporation into CIII decreases with increasing age, the detectable small amount of fully assembled CIII was bound to SC1. The respiratory chain supercomplexes contained less RISP protein but equal amounts of Core1 subunit and CI as controls, indicating that CIII was present as a pre-CIII. This did not affect the SC1 stability. Neither was the function of CI nor that of CIV compromised. In fact, CI activity was increased as shown in the respirometry assay using the malate/pyruvate substrate. As a result of increased protein degradation glutamate is increased in sick homozygotes [25], which explains why addition of glutamate in the respirometry did not result in a significant increase in oxygen consumption. The low response to CII substrate indicates mitochondrial deficiency in *Bcs1l^G/G^* under functional stress due to convergent electron input [15]. CI in supercomplexes was fully assembled as in controls, as shown by the presence of the NADH dehydrogenase subunit NDUFV1 [23] in SC1. In addition, free fully assembled CI was abundant in homozygotes.

Our results support those of a study on RISP knockout mouse fibroblasts [24], in which supercomplex formation was addressed both when cells were subjected to hypoxic (1% oxygen) and hyperoxic (20% oxygen, i.e. hyperoxia compared to normal organ oxygen tension) conditions. Destabilization of respiratory chain complexes occurred only in association with increased ROS (i.e. 20% oxygen), whereas normal levels of complexes and supercomplexes were present during hypoxic conditions. The authors concluded that supercomplexes and CI are disassembled under conditions of elevated ROS both in wild type and RISP knockout cells. In a metabolomics study of *Bcs1l* ^G/G^ mouse liver tissue, we found slightly increased indicators of ROS only at end-stage disease, P29-P30 [25]. In the present study there was no disassembly of supercomplexes in the P27–29 animals, suggesting that ROS probably had a minor effect on supercomplex assembly. A recent study on interaction between supercomplexes and ROS showed that supercomplex formation in fact may limit production of ROS [7].

The effect of deficient CIII assembly on supercomplex formation has been studied in a few other models. In mitochondria of human skeletal muscle cells with mutations in the mitochondrial encoded cytochrome b subunit of CIII, the severely but not completely reduced CIII resulted in prevention of supercomplex formation and decreased stability of CI [4]. In *Bcs1l* ^G/G^ mice, precomplex of CIII was abundant and some correctly assembled CIII was present, which may account for the absence of such profound changes.

In a cultured tumor cell model, where all complexes with mitochondrial encoded subunits were down regulated with doxycycline, the effects of individual complexes were investigated based on mitochondrial DNA recovery after the treatment [14]. The results suggested that a CI assembly intermediate incorporates all CIII and CIV subunits and when these complexes are fully assembled the NADH dehydrogenase module is added to CI as a final step [14]. Both CIII_2_ and CIV were present in free form but fully assembled CI was only detected in the respirasome. In line with that, we found in wild type animals fully assembled CI mainly in supercomplexes. In mutant homozygotes, however, fully assembled CI including NDUFV1 was present both in free form and in supercomplexes together with pre-CIII and fully assembled CIII. Whether free CI is a result of increased release from the supercomplex or increased assembly of CI without incorporation into respirasomes with complexes III and IV cannot be concluded from this study. In any case, the deficient CIII assembly compromised neither CI stability nor function in *Bcs1l* ^G/G^ mice. On the contrary, there was an increased expression of CI subunits and increased CI activity revealed by CI oxygen consumption in respirometry. Thus in our study CI was not dependent on fully assembled CIII, as reported in doxycycline-treated cells [14]. The difference may be ascribed to the different models used. In the cell culture model, doxycycline depletes through inhibition of translation all mitochondrial encoded proteins including CYTB, which has been proposed as the nucleating unit for CIII assembly in yeast [26]. Furthermore, doxycycline treatment causes a partial loss (ca 40%) of mitochondrial DNA [14]. As absence of mitochondrial DNA is associated with down regulation of nuclear encoded subunits of CIII [27], doxycycline administration probably caused many cellular changes including Core1 and RISP down regulation in addition to the desired ones [14]. In our *in vivo* model, the *Bcs1l* mutation results in diminished levels of BCS1L protein in all tissues [15]. This progressively impairs incorporation of RISP protein into CIII leading to an accumulation of pre-CIII that can subsequently associate with other complexes to form supercomplexes. The impairment of BCS1L function and diminished RISP amount did not result in overexpression of *Bcs1l* mRNA, nor of RISP or other CIII subunits.

Our data, like those in hypoxic RISP knockout cells [24], indicate that pre-CIII_2_ can interact with CI in a pre-CIII_2_/CI supercomplex. This interaction might be stabilized by CIV, concluded from the finding that free CIV was elevated in sick *Bcs1l* ^G/G^. A similar situation was described in tissues from NDUFS4 knockout mice in which CI lacking NDUFS4 was stabilized by associating with CIII, thereby enabling full assembly of CI in the respirasome [28]. Whether the small amount of correctly assembled CIII is crucial for supercomplex formation and complete lack of RISP would prevent it, cannot be investigated in our model because homozygotes do not survive to that stage. The lack of CIV in SC1 can be ascribed to the genetic background in C57Bl/6 mice being homozygous for the short form of supercomplex assembly factor I (SCAFI) resulting in a main supercomplex containing CI and CIII, but no CIV [12].

In conclusion, our study on supercomplexes in *Bcs1l* ^G/G^ mitochondria demonstrates that the supercomplex assembly in tissue can be modified depending on CIII assembly deficit. A recent publication showed in detail the dynamics of supercomplex assembly [12]. Structural or functional disturbances in the respiratory chain can be compensated by altered supercomplex assembly, thereby ensuring sufficient respiratory chain activity. Such a compensatory mechanism in *Bcs1l* ^G/G^ mitochondria is supported by the finding of a preserved respiratory chain function until it is forced to maximal capacity. Whether the compensatory mechanism varies between tissues and thus plays a role in tissue specificity of mitochondrial disorders due to CIII deficiency is unclear and warrants further study.
